# Assessment of CEPH-Accredited Institutions Offering Public Health Programs in the United States: A Short Report

**DOI:** 10.3389/fpubh.2015.00290

**Published:** 2016-01-27

**Authors:** Ashish Joshi, Chioma T. Amadi

**Affiliations:** ^1^City University of New York, New York, NY, USA; ^2^City University of New York School of Public Health, New York, NY, USA

**Keywords:** public health, programs, masters, doctoral, education

## Abstract

**Aims:**

Examine the distribution of the Council of Education in Public Health (CEPH)-accredited institutions offering public health educational programs in the United States, and characterize their various attributes.

**Methods:**

A search was conducted during the period of June 2014, using the Association of Schools and Programs of Public Health database (ASPPH), and individual university websites to obtain a complete list of CEPH-accredited institutions offering programs in public health at the Certificate, Masters, and Doctoral levels in the United States. Detailed information were abstracted from the various programs offerings, including school/program information, school type, geographic location, admission cycle, education delivery format, public health concentration, number of credits, presence of a global component, joint programs, and tuition. These data were analyzed in August 2014.

**Results:**

A total of 85 CEPH-accredited institutions designated as either “Schools of Public Health” or individual “Programs of Public Health” were present in the ASPPH database at the time of this data collection (2014). These institutions offer programs in public health at the Certificate (61%, *n* = 52), Masters (100%, *n* = 85), and Doctoral (44%, *n* = 37) levels in the United States. More than half of the programs offered were provided by schools of public health (58%, *n* = 49), which were mostly public universities (75%, *n* = 64), concentrated in the Northeast (22%, *n* = 19) and mainly admitted students during the fall semester. Ninety-three concentrations of public health currently exist, of which 25 concentrations are predominant.

**Conclusion:**

To the best of our knowledge, this is the first study that examines the distribution of existing CEPH-accredited public health educational programs offered by United States institutions. We suggest future areas of research to assess existing public health workforce demands, and map them to the curriculums and competencies provided by institutions offering public health educational programs in the United States. This could provide valuable insight on the extent to which public health curriculums are meeting workforce demands.

## Introduction

Public health has been described as “the science and art of preventing disease, prolonging life, promoting health and well-being through organized community efforts for the sanitation of the environment, control of communicable infections, organization of medical and nursing services, early diagnosis and prevention of disease, education of the individual in personal health, and the development of the social machinery to assure everyone a standard of living adequate for the maintenance or improvement of health” (1). The advent of public health can be traced back to the emergence of infectious disease epidemics, and the current state of natural biological threats to humanity poses a constant challenge to the capacity of the health workforce in containing them (2). Efficient public health systems are invaluable in assessing disease burden, responding to outbreaks, evaluating existing strategies, implementing public health initiatives, and generating evidence-based interventions to health problems (3, 4).

Achieving global health goals is contingent on health systems strengthening through assuring a growing competent public health workforce (3). Public health professionals constitute individuals trained in a variety of occupational settings and could include physicians, engineers, statisticians, educators, or even communication experts since the broad scope of public health requires a diverse workforce of professionals (5, 6). However, majority of these individuals may have not acquired formal training in public health, but they possess an array of public health expertise from their disparate work domains. This creates the need for innovate approaches in public health training to harness and cater to the emerging workforce needs (6). Hence, the relevance of academic institutions, types of programs, specific program concentrations and other attributes are critical in assuring an informed workforce (6).

The educational curriculum utilized by schools offering training programs in public health is one which incorporates primary training in the five basic core sciences, including epidemiology, biostatistics, behavioral and community health, environmental health, and health services administration (6). In addition, the past decade has witnessed an evolution in public health training programs evidenced by the incorporation of novel disciplines, including health informatics, nutrition, genomics, community-based participatory research, global health, and law and ethics into the public health curriculum (6–10). Consequently, the workforce demand for these emerging programs are on the increase, highlighting the need for tailored approaches that incorporate these novel fields into the existing curriculum to ensure that training programs are meeting the workforce demands (7–10). The objective of this short report is to examine the distribution of the Council of Education in Public Health (CEPH)-accredited public health educational programs offered in the United States, and characterize their various attributes.

## Materials and Methods

A search was conducted during the period of June 2014, using the Association of Schools and Programs of Public Health database (ASPPH), and individual university websites to obtain a complete list of CEPH-accredited institutions offering programs in public health at the Certificate, Masters, and Doctoral levels in the United States. Detailed information regarding each public health program were abstracted from the various programs, and they included (a) school/program information; (b) school type (public/private); (c) geographic location of the school (North, South, West, East, Northwest, Northeast, Southwest, Southeast, and Central); (d) admission cycle (fall/spring/summer or a combination); (e) education delivery format (online/on-campus/both); (f) public health concentration at Certificate, Masters, and PhD levels; (g) number of credits; (h) presence of a global health concentration (yes/no); (i) joint degrees (yes/no) and type; and (j) tuition costs (cost per credit/cost per semester/cost per annum). The schools were contacted directly through phone calls and emails to obtain complete information, including admission cycle, mode of delivery, or duration of the public health programs offered, which were not clearly stated on their websites. No IRB was needed for this study as the data gathered was publicly available information on the school websites, the schools were only contacted for clarity purposes. Also, no individual-related information was gathered. These data were analyzed in August 2014.

### Statistical Analysis

Descriptive analysis was conducted for all variables. Results of all categorical variables are presented as frequency distributions. All statistical analysis was performed using SAS version 9.1.

## Results

### School/Program Information

**Figure 1:**
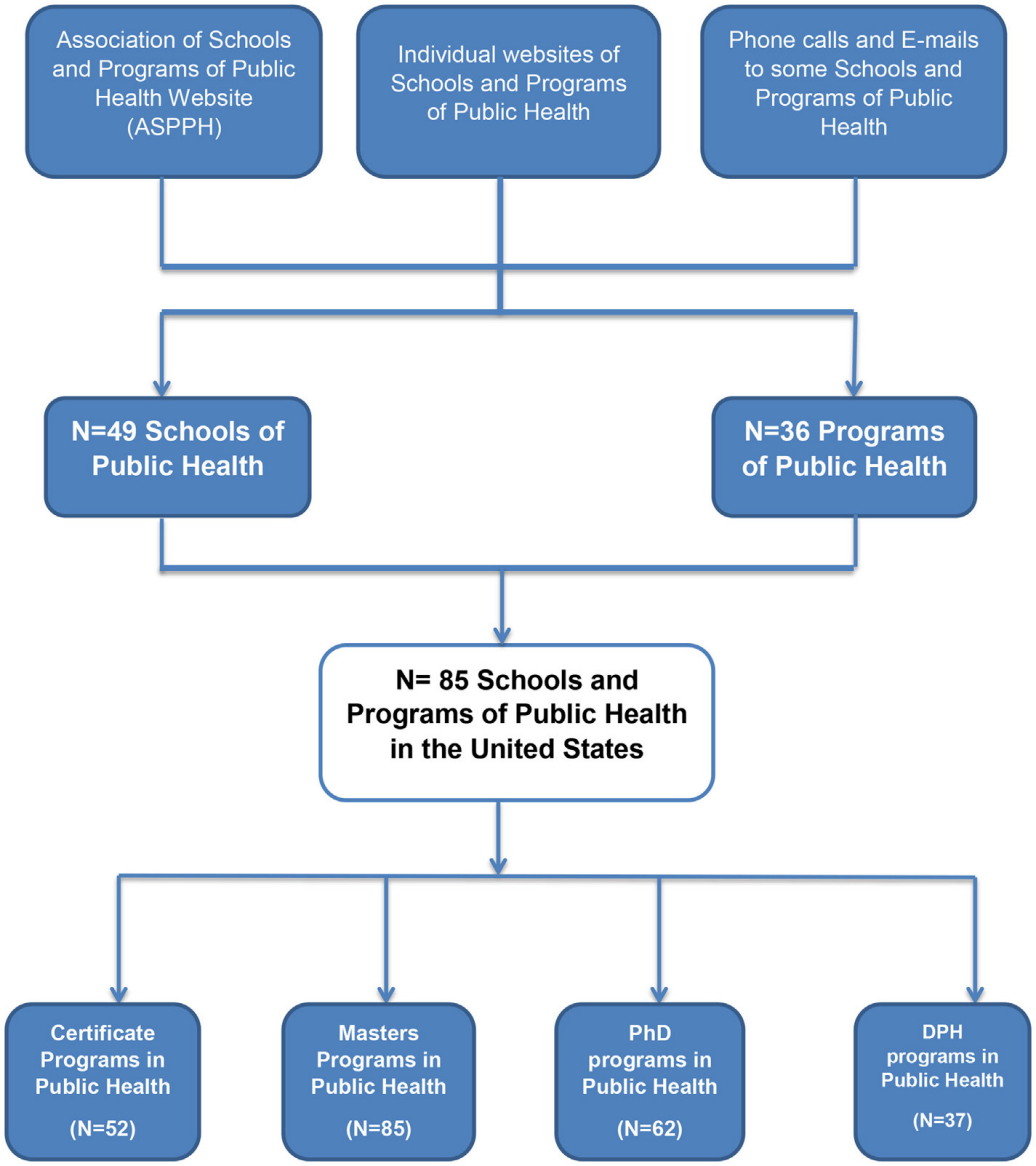
**Schools and programs of public health in the United States**.

A complete list of 85 CEPH-accredited institutions designated as either “Schools of Public Health” (58%, *n* = 49) or individual “Programs of Public Health” (42%, *n* = 36) offering public health programs at the Certificate, Masters, and Doctoral levels in the United States were present in the ASPPH database at the time of this data collection (Figure 1).

### School Type (Public/Private)

More than half of the training programs are offered by schools of public health (58%, *n* = 49), and mainly from public universities (75%, *n* = 64).

### Geographic Location

Majority of these schools offering Certificate, Masters, and PhD/DPH programs are located in the Northeast (22%, *n* = 19) followed by East (16%, *n* = 14), South and Central (14%, *n* = 12), and West (12%, *n* = 10).

### Admission Cycle

Applications were mostly accepted during the fall semesters exclusively, at the Masters (58%, *n* = 49), PhD (88%, *n* = 55), and DPH (86%, *n* = 32) levels, whereas certificate programs were more commonly offered during both fall and spring semesters (34%, *n* = 18).

### Education Delivery Format

The Certificate programs commonly had both online and on-campus offerings inclusively (42%, *n* = 22), compared to the Masters (33%, *n* = 28), or PhD and DPH (8%, *n* = 3) programs. All the PhD programs were offered on-campus only, compared to the DPH programs where 3% were offered as online programs.

### Public Health Concentration at Certificate, Masters, and Doctoral Levels

**Figure 2:**
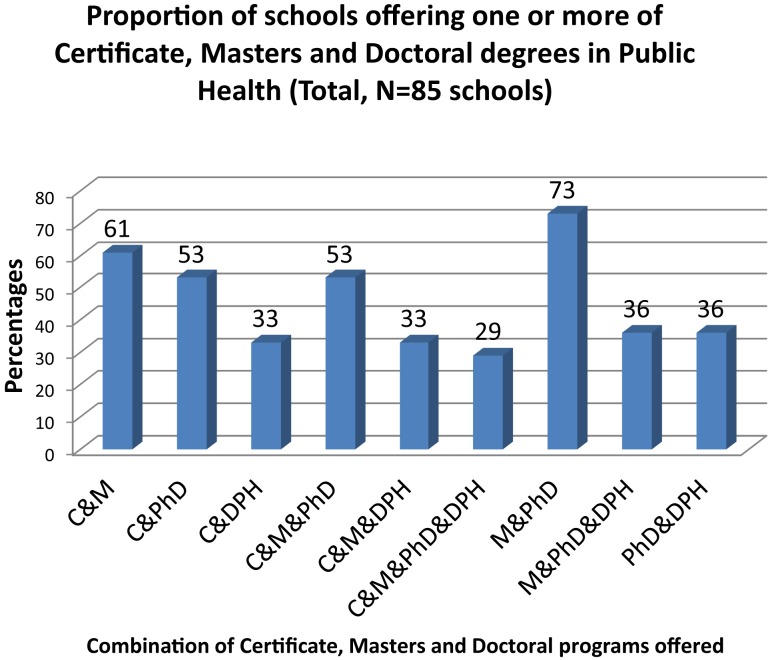
**Proportion of schools offering one or more of Certificate, Masters, and Doctoral degrees in public health**. C, certificate; M, masters; PhD and DPH, doctoral degrees; “&,” “and.”

Hundred percent (*n* = 85) of the institutions examined offer Masters of Public Health (MPH) programs, followed by PhD programs (73%, *n* = 62), Certificates (61%, *n* = 52), and DPH programs (44%, *n* = 37). Among the programs offered, combined Masters and PhD (73%, *n* = 62) were most common, followed by combined Certificate and Masters programs (61%, *n* = 52) (Figure 2). There are currently 93 concentrations of public health at the Certificate, Masters, and Doctoral levels in the United States, of which 25 concentrations are predominant. Social Behavioral Health Sciences (75%; *n* = 64), Epidemiology (71%; *n* = 60), Environmental Health (69%; *n* = 59) and Health Policy Management/Health Policy (63%; *n* = 53), and Biostatistics (61%; *n* = 52) were the most common MPH concentrations, and are also the core public health concentrations defined by the Council of Education on Public Health (12).

○ *Certificate Programs in Public Health*: 61% (*n* = 52) of the schools offered certificate programs in public health. More than half of the certificate programs are delivered as face-to-face (52%, *n* = 27), whereas 42% (*n* = 22) are delivered as a combination of both face-to-face and online delivery. The most common public health concentrations at the certificate level included general public health (20%, *n* = 17) followed by global health (12%, *n* = 10).
○ *Masters Programs*: 100% (*n* = 85) of the institution examined offered Masters in public health. The most common Masters programs are listed in Table 1.
○ *Doctoral Programs in Public Health (PhD/DPH)*: majority of the doctoral programs offered in schools and programs of public health are PhD programs (73%, *n* = 62), compared to DrPH programs (44%, *n* = 37). The most commonly offered PhD program in public health is in the Epidemiology concentration (49%, *n* = 42), while the concentration Social and Behavioral Sciences is more commonly offered as a DrPH program (39%, *n* = 33).

**Table 1:** **Distribution of the most common Master’s degree concentrations offered across schools and programs of public health in the United States**.

Master’s degree in public health: concentrations offered	Frequency, *N* (%)
Public health nutrition	12 (14)
Maternal and child health	13 (15)
Health-care management	13 (15)
Health services research and administration/health administration	15 (18)
Global health	15 (18)
General public health	24 (28)
Biostatistics	52 (61)
Health policy/health policy and management	53 (62)
Environmental health	59 (69)
Epidemiology	60 (71)
Socio-behavioral health sciences	64 (75)

### Number of Credits

The average course credits for most of the certificate programs vary across the schools, ranging from about 12 to 35 credit units, which often consist of 4–6 courses that can be completed within a semester of study. Most of the master’s programs require an average of 42 credits of coursework for completion, with others ranging between 39 and 80 credits. DPH programs frequently required between 30 and 121 credits for completion, whereas PhD programs required between 42 and 120 credits.

### Presence of a Global Health Concentration

Public health programs with a global health concentration are offered only among 35% (*n* = 30) of the institutions, predominantly at either Certificate (12%, *n* = 10) or Masters (18, *n* = 15) levels. Only 29% (*n* = 25) of the institutions offer programs at the Certificate, Masters, and Doctoral levels inclusive. Majority of the global health programs are located in the East (9%, *n* = 8), followed by West (7%, *n* = 6) and the North east (7%, *n* = 6) regions of the United States.

### Joint Degree Programs

Sixty-six percent (*n* = 56) of the schools offered one or more joint or dual MPH degrees, often in combination with common programs, including Social Work: MPH/MSW (25%, *n* = 21), Medicine: MPH/MD (48%, *n* = 41), Business Administration (22%, *n* = 19), Law: MPH/JD (34%, *n* = 29), Nursing: MPH/MSN (12%, *n* = 10), and Pharmacy: MPH/PharmD (8%, *n* = 7).

### Tuition Costs across Public Health Programs in the United States

Tuition costs are reported by 83 schools and programs of public health in the United States in several formats, including cost per credit (72%, *n* = 60), cost per semester (8%, *n* = 7), and cost per annum (19%, *n* = 16). More than half of the schools (72%, *n* = 60) report their cost of tuition per credit hour. Majority of the in-state costs per credit hour are in the range of $300–$599 followed by a range of $600–$899 (15%, *n* = 9) for schools reporting tuition costs per credit hour (*n* = 60). Out-of-state tuition costs per credit hour are mainly between the range of $900–$1,199 (27%, *n* = 16). In-state and out-of-state costs are mainly in the range of $4,000–$15,999 for schools reporting tuition costs per semester (*n* = 7). For schools reporting tuition per annum (*n* = 16), in-state and out-of-state costs are in the range of $20,000–$24,999 (37%, *n* = 6) and $30,000–$34,999 (37%, *n* = 6).

## Discussion

There is a growing need for public health professionals in diverse fields of practice in the twenty-first century (5). Prior research has emphasized the absence of formal public health education and training among the majority of the current workforce (6, 11). Results of our study showed that 85 schools and programs currently offer professional training in public health at the Certificate (61%, *n* = 52), Masters (100%, *n* = 85), and Doctoral (44%, *n* = 37) levels in the United States. There indicates a need for more online certificate programs in public health that can provide short-term formal training in public health for the current workforce, by providing them timely skills that are needed to address the various public health challenges.

Our findings show that 93 concentrations of public health currently exist at the Certificate, Masters, and Doctoral levels in the United States, of which 25 concentrations are predominant. Social Behavioral Health Sciences (75%, *n* = 64), Epidemiology (71%, *n* = 60), Environmental Health (69%, *n* = 59) and Health Policy Management/Health Policy (63%, *n* = 53), and Biostatistics (61%, *n* = 52) are the most common concentrations, and are also the core public health concentrations defined by the Council of Education on Public Health (12). Asides from these core concentrations, numerous public health concentrations are evolving along with their associated competencies (5, 6). However, we identified significant overlaps in the nomenclature of similar public health concentrations across the schools offering these programs. As a result, prospective students in the field of public health are potentially faced with a plethora of choices of public health concentrations with the same objectives and competences across different educational Institutions. This key challenge needs to be addressed by ensuring clear distinction on the objectives of the various public health program nomenclatures.

The current state of evolution and diversity of the existing public health concentrations in the twenty-first century now creates even more extensive educational needs (5). The relevance of specific public health concentrations, including public health informatics, global health, communication, and leadership, is increasingly recognized. Specifically, the role of Informatics to both public health and health services delivery has been increasingly recognized over the past decade, as evidenced by a rapid increase in electronic storage and exchange of health information, increase in health-related datasets, and the need for global innovative Informatics solutions (8). Global perspective needs to be incorporated into the interdisciplinary nature of public health to ensure that professionals are competent in working together as globally connected teams; hence, the need for more evidence-based Global Health training programs (9). Results of our analysis have shown that limited programs exist in these key areas. Current public health practice demand for these specific concentrations is on the increase, suggesting a need for a wider array of flexible training programs in these areas (13).

In addition, existing literature has identified a disconnection between Nutrition and Dietetics workplace demands and the content of existing educational curriculums and learning objectives taught across nutrition programs (14, 15). Speculations that the same scenario may apply to public health programs exist, suggesting the need to examine the existing public health workforce demands and identify the extent to which they are being met by the existing curriculums utilized by schools and programs of public health.

### Limitations

A limitation of the current study is the possible recent inclusion of new public health programs to the existing programs in various institutions, from the time the data search was conducted in 2014, till present. In addition, the restriction of the current study to post-bachelor educational programs is also a relevant limitation of this study, and a suggested future area of research.

## Conclusion

To the best of our knowledge, this is the first study that examines the distribution of existing CEPH-accredited public health educational programs offered by United States institutions. We suggest future areas of research to assess existing public health workforce demands, and map them to the curriculums and competencies provided by institutions offering public health educational programs in the United States. This could provide valuable insight on the extent to which public health curriculums are meeting workforce demands.

## Author Contributions

All authors listed contributed to the manuscript preparation and review. AJ developed the manuscript outline and objectives, guided the writing of the other sections, and proof read the final manuscript. CA drafted the initial version of the manuscript, collected and analyzed data, and made corrections to the final copy.

## Conflict of Interest Statement

The authors declare that the research was conducted in the absence of any commercial or financial relationships that could be construed as a potential conflict of interest.
